# The teachers who leave: Teacher attrition in Burkina Faso

**DOI:** 10.1016/j.ijedudev.2025.103228

**Published:** 2025-03

**Authors:** Biniam Bedasso, Amina Mendez Acosta

**Affiliations:** a Center for Global Development, 1 Abbey Gardens, Great College St, London SW1P 3SE, United Kingdom; b Consultant for Center for Global Development, 2055 L St NW, Washington, DC 20036, United States

**Keywords:** Education, Teachers, Attrition, Low- and middle-income countries

## Abstract

High teacher attrition affects education systems through direct costs in replacing teachers who left the service, and indirect costs in classroom disruption and loss in experience. Efforts to address teacher shortage must be informed by which teachers leave and why. Using administrative data from Burkina Faso, we aimed to analyze the demographic and geographic correlates of teacher turnover. We find that early career teachers, female teachers, and teachers with tertiary education, are more likely to attrite. Teachers who hold higher positions—such as school principals—have better retention rates. In terms of school-level attrition, rural and remote schools tend to lose teachers to other schools whereas schools in urban or more developed regions are more likely to lose teachers to options outside of the teacher workforce. Finally, we discuss policy options in improving teacher retention given these findings.

## Highlights

• Early-career teachers, female teachers, and those with tertiary education are more likely to leave the profession.
• School principals and teachers in urban areas are retained longer than those in rural or remote schools.
• Urban schools lose teachers to opportunities outside of teaching, while rural schools lose teachers to urban schools.
• Teachers in conflict-affected areas are more likely to leave the profession.

## 1 Introduction

Government efforts to recruit teachers and improve the quality of teaching in low- and middle-income countries (LMICs) can be undermined by high teacher turnover. Almost half of the education budget in LMICs go to teacher wages (Crawfurd and Pugatch, 2022). High teacher attrition represents significant loss in resources by way of high costs of recruiting and training replacement teachers. Even when teachers are quickly recruited to replace those who exit the service, it might take considerable time from recruitment to deployment. This means classroom activities and academic calendars are affected, which leads to loss of instructional time, or the new teachers might not have the same level of skills and experience as those who choose to leave (UNESCO, 2010). In these scenarios, student learning may suffer. For example, a teacher resigning in Rwanda is associated with lower school test scores, potentially driven by an increase in class size and the high share of teachers who provide instruction outside of their subject matter expertise (Zeitlin, 2021).^1^ These impacts are potentially bigger on poorer and more remote schools, which already experience challenges in recruitment and retention and where quality teachers are already in short supply (Evans and Mendez Acosta, 2023b).

Improving teacher retention is particularly important in the context of the increasing demand for teachers expected in the coming years driven by population growth and improving enrollment rates. In Sub-Saharan Africa, countries would need to recruit 15 million teachers to meet the Sustainable Development Goal 4, i.e. education for all at the primary and secondary level by 2030 according to a UNESCO report (2024). From the same report, about one-third of this number (5.6 million teachers) will be needed to fill vacant posts due to attrition while the rest will be needed to fill new positions. This reflects the rapidly growing youth population in the region which is expected to grow by 1.5 times between 2022 and 2040, compared to 1.2 times in South Asia based on authors’ analysis using data from the UN Population Division (2022). In contrast, the population growth rates in other regions such as East Asia and the Pacific and Europe and Central Asia are expected to remain stagnant for the same period (UN Population Division, 2022). Efforts to meet this teacher demand will require better teacher hiring and deployment practices, and better retention of teachers through better teacher working conditions. Understanding the profile of teachers who leave the workforce and where in their career trajectory they are likely to leave can help shape policies to prevent early exit from the teacher workforce.

Teachers may exit the work force earlier than the retirement age due to a combination of better opportunities outside of teaching (such as other civil service positions or jobs in the private sector), dissatisfaction with their working conditions (poor pay, remote assignments, or limited classroom resources), demographic and personal factors (such as illness or to raise a family), or combinations of such factors (for example, how a teacher’s race or caste fits the social and cultural dynamics of the school they were assigned) (UNESCO, 2010, Nguyen et al., 2020). Governments have sought to address these factors through different policies. One of type these policies provides incentives to stay in the profession such as financial incentives implemented in Latin America (Cabrera and Webbink, 2020, Camelo and Ponczek, 2021; Hinze-Pifer and Méndez, 2016) and in Sub-Saharan Africa (Chelwa et al., 2019, Pugatch and Schroeder, 2014, Pugatch and Schroeder, 2018, Swai, 2013). Another set of policies are national recruitment drives such as the ones recently implemented in Tanzania, Kenya, and Zimbabwe (Byemelwa, 2024, Moses, 2024, Ziana, 2024). Yet another type of policies targets specific groups of teachers such as female teachers (Gad, 2015, Kamere et al., 2019).

In this paper, we attempt to examine the demographic profiles of teachers who leave the profession and the predictors of school-level attrition in Burkina Faso. As a low-income country that has been struggling to maintain access to education and improve quality amid chronic poverty and widespread conflict, Burkina Faso could serve as a good case study for a context in which teachers might be faced with economic hardships, insecurity, and limited outside options in deciding to stay in their jobs. As such, gaining a better understanding of the individual characteristics of teachers who are more likely to leave and the type of schools that are more likely to experience higher attrition could be useful to devise better targeted retention strategies.

Examining teacher retention trends requires panel data on teacher movement in and out of the workforce and between schools as well as demographic and location-specific characteristics. We use the School Census Data from Burkina Faso’s Education Management Information System (EMIS) from 2014 to 2019 to build the attrition profiles of teachers with respect to various demographic and socioeconomic factors. We also compute the school-level rates of attrition consisting of teachers who leave the profession and those who transfer to another school. We find that teachers in the first five years of teaching, teachers with university degrees, and female teachers are more likely to leave the teaching profession. This suggests that competing professional interests outside the teacher profession and personal milestones such as childbirth and domestic responsibilities may motivate attrition.

At the school level, we find that there is a significant difference between factors that are associated with the number of teachers that leave the profession and those who switch schools. Specifically, schools that are located in urban areas and more developed regions are likely to experience more attrition in terms of teachers leaving the profession for outside options whereas schools in rural areas and less developed regions are more prone to teachers transferring to urban schools. We also summarize the policy options and their available evidence in improving teacher retention such as providing monetary incentives and similar benefits, enhancing opportunities for teacher professional development, improving overall job satisfaction, and addressing specific barriers that female teachers face. We highlight how the policies may be relevant to the Burkina Faso context based on our findings on the correlates of attrition.

We add to two bodies of literature and present research from a new data source. First, we contribute to the general understanding of the challenge of teacher attrition in the Sub-Saharan Africa region, especially on the demographic and geographic determinants of the decision to leave the workforce. Second, we summarize the existing evidence on policies that improve teacher retention in LMICs and highlight the interventions that are promising but do not have sufficient evidence. Finally, we contribute to the literature using a novel data source (teacher panel data) from a developing country in armed conflict facing multiple challenges in teacher retention (Burkina Faso) that has not been previously used to provide insights to teacher attrition.

The remainder of the paper is organized as follows: Section 2 provides the context on teacher attrition in LMICs and the teacher working conditions in Burkina Faso, Section 3 discusses the methodology and its limitations, Section 4 reports the results, Section 5 discusses some of the policy options, and we conclude in Section 6.

## 2 Global and local context

### 2.1 Teacher retention in low- and middle-income countries

**Figure A1:**
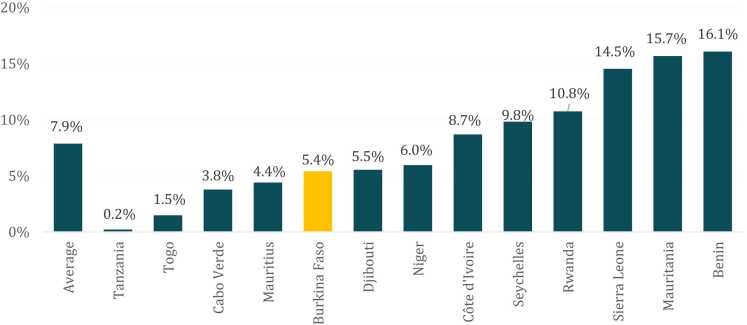
Teacher attrition rates among primary school teachers in several countries in Sub-Saharan Africa (2018–2022), Source: UIS Statistics (UNESCO, 2020), Note: These are the only countries from the region with teacher attrition rates reported in the database for the years 2018–2022

Retaining teachers, especially quality teachers, is a challenge for many education systems and particularly so in Sub-Saharan Africa (Mulkeen et al., 2007; Pitsoe and Machaisa, 2012) (UNESCO, 2023). Earlier work describes a “hemorrhage of teachers who leave the profession before retirement age” in a paper that reports on efforts to retain secondary level teachers in Sub-Saharan Africa (Mulkeen et al., 2007) while another paper refers to the “teacher attrition catastrophe” in the region. A more recent work shows lower attrition levels but highlight structural inefficiencies: an analysis of 30 countries in Sub-Saharan Africa region shows the average attrition rate for primary school in the mid-2010s was 4.8 percent (Bennell, 2023). Even with lower attrition, the delays in replacing these teachers, which in some cases may take years, drive the high vacancy rates and amplifies the impact of teacher attrition on actual availability of teachers ready to teach in classrooms. The latest attrition rates show a higher average of 8 percent between 2018 and 2022 across thirteen countries (Appendix Figure A1). Some countries in particular struggle with relatively high teacher attrition: Rwanda, Sierra Leone, Mauritania, and Benin all have attrition rates above 10 percent. Burkina Faso is close to the median with an attrition rate of 5.4 percent for the period. The challenge of retaining teachers is not unique to low- and middle-income countries: the average teacher attrition rate in 14 OECD countries in 2016 is at 6.6 percent (OECD, 2021). However, the stronger education systems in these countries allow for quicker recruitment and deployment such that effects on student learning and participation might be better managed and mitigated.

Why do teachers leave? Teachers may voluntarily leave before reaching retirement age due to demographic and personal factors (such as illness or to raise a family), more attractive opportunities outside teaching either through better paid civil service positions or other non-teaching jobs in the private sector, concerns on career development and progression, or other issues relating to teacher working conditions (UNESCO, 2010, Nguyen et al., 2020). In Cameroon, migration of new teachers to other government departments is only next to retirement and death in top reasons cited for teacher attrition (World Bank 2018a). Similarly in Guinea-Bissau, many teachers leave to join other ministries after the first year of teaching, and as many as 90 percent of teachers in public schools also work in the private sector as their second job (World Bank, 2018b, World Bank, 2019).

Not many studies attempt to profile the type of teachers who leave, potentially because of the limitations on data availability. One study that does so uses administrative records and teacher placement data in Rwanda to identify the profile of teachers who attrite (Zeitlin, 2021). Male teachers, teachers of Math subjects, and those in their first five years of tenure are significantly more likely to leave the profession, potentially because of better job opportunities outside teaching available to young professionals with strong quantitative background. Teacher attrition in Rwanda does not seem to be driven by rural-urban designation, but attrition is surprisingly negatively associated with pupil-teacher ratio (such that teachers are less likely to leave schools with higher class size) which could be the result of both teachers and students sorting to better schools over time.

Several other studies — albeit of more qualitative nature — show both personal factors and lack of resources as drivers of teacher attrition. A survey of about 100 current and former secondary school teachers in Kenya shows that male teachers are more likely to attrite, and that unlike in Rwanda, married teachers and teachers with work experience of over 20 years are more likely to quit (Mabeya 2021). In South Africa, qualitative surveys with a small sample of teachers cite unfavorable working conditions due to “disintegration of discipline,” limited facilities for teaching, inadequate incentives, and school overcrowding as detriments to teacher retention (Pitsoe 2013). Earlier studies suggest poor teaching conditions in Senegal, Burkina Faso, and Mali, including classroom overcrowding, shortage of textbooks and teaching materials, and subpar living accommodations lead to teachers quitting (Caillods 2011; Mulkeen et al., 2007).

There is also a rich literature on teacher attrition in high-income contexts: a meta-analysis found 120 studies that report on associations of personal and school-level correlates to teacher turnover (Nguyen et al., 2020). Although none of the papers use data from low- or middle-income countries, the review presents a conceptual framework for understanding teacher attrition that is still relevant to the context of developing countries. The framework combines three main drivers: (1) personal factors such as demographics and teacher qualifications, (2) school factors such as location, resources, and learners’ profiles, and (3) external policy factors such as pay and evaluation policies, presence of unions and hiring practices. Similarly, there is a rich adjacent literature (including a systematic review of over 100 studies) on improving retention of health workers especially in rural and remote areas that parallel many of the findings on why teachers leave the profession and what might work to retain them (World Health Organization, 2020). For these civil servants, isolated initiatives such as short-term financial incentives rarely work or only work while the incentives are in place, but the effect quickly fades after the end of the program. Rather, monetary and non-monetary incentives delivered with initiatives that improve community and family engagement, wider health services reform. and more sustainable service redesigns together have a higher likelihood of affecting change.

Countries experiencing active armed conflicts are particularly vulnerable to teacher shortages. Human Rights Watch (2020) documented 126 attacks on schools, students, and teachers in Burkina Faso between 2017 and 2020, and over 270 attacks in 2022–2023 (Global Coalition to Protect Education from Attack 2024). Aside from direct threats to physical safety to students and education professionals, a conflict-induced crisis also disrupts critical education infrastructures such as disbursement of salaries, professional development activities, school construction, and supplies of teaching materials (Mendenhall et al., 2018). Protracted crises also affect longer-term supply of teachers (disrupted education will produce fewer and less-qualified teachers) and education financing (limited national budgets competing against rebuilding and rehabilitation, and short-cycled humanitarian funding being incompatible to sustaining recurring teacher salaries costs) (Nicolai and Hine 2015). Migration adds another layer of complexity when refugees settle in areas already facing teacher shortages and overcrowding, teachers lose their work permits when crossing borders, and teachers preemptively migrate away from conflict areas (UNESCO 2018).

While demographic patterns might be influenced over time through targeted hiring practices, governments and policy actors can also explore more immediate and sustainable ways to improve teacher working conditions in general to encourage the best of teachers in the workforce to stay.

### 2.2 Teacher working conditions in Burkina Faso

Some of the factors to attrition mentioned above might be more applicable to Burkina Faso than others. An analysis of teacher pay in 15 countries in Sub-Saharan Africa shows that the monthly median earnings of teachers in Burkina Faso of 650 dollars per month (PPP in 2011 dollars) is comparable to peers in the region where the average is 677 dollars per month, ranging from 100 dollars in Democratic Republic of the Congo to 2306 dollars in Namibia (Evans et al., 2022). Teacher salary in the country is almost three times the average of other wage workers despite working up to 25 percent fewer hours than the same wage workers (40 hours versus 53 hours per week). However, as with any other profession, opportunity cost increases as individuals get more qualified. Teachers with secondary education earn as much as other managers and other professionals, but teachers with post-secondary education can earn twice more by switching sectors. Similar to many countries, teachers with permanent contracts earn much more than teachers with temporary contracts; in Burkina Faso, civil service teachers on average earn almost three times that of contract teachers.

Teacher deployment is rigid with limited inter- and intra-region mobility (UNESCO, 2020). Teachers declare the region they intend to work in during the application process to be certified teachers. Once hired, they are deployed within the region they selected based on which schools experience the highest teacher shortages. They may request to transfer to a different school within the region after three years of service, and to transfer to a school outside of their region after six years. Majority of new teachers are often assigned to rural locations in favor of putting teachers with more seniority in more desirable locations (Sirois, 2017).

**Figure A2:**
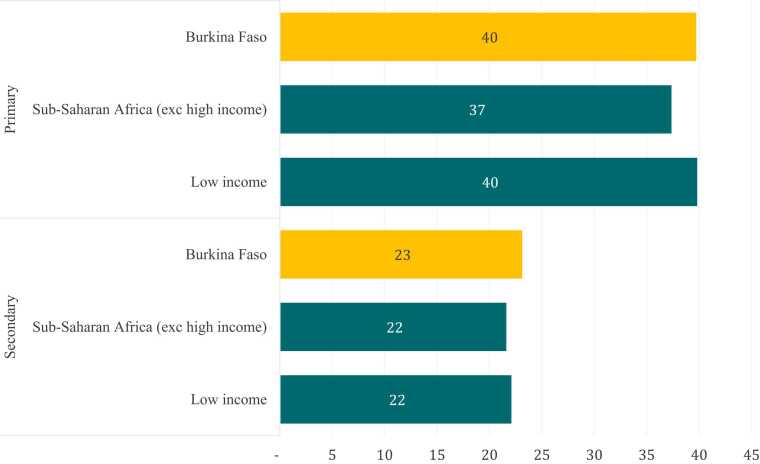
Pupil-teacher ratios at the primary and secondary level for Burkina Faso, low- and middle- income countries in Sub-Saharan Africa and low-income countries (2018 rates), Source: World Bank Development Indicators (World Bank, 2023)

**Figure A3:**
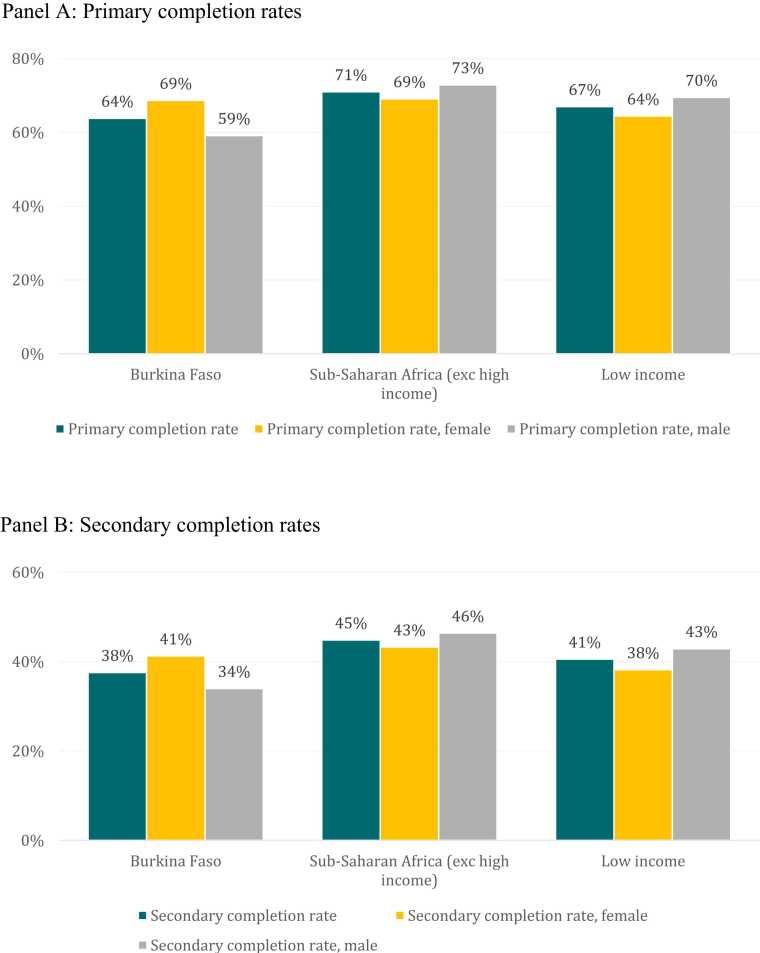
Completion rates for Burkina Faso, low- and middle- income countries in Sub-Saharan Africa and low-income countries (2021 rates), Panel A: Primary completion rates., Panel B: Secondary completion rates, Source: World Bank Development Indicators (World Bank, 2023)

The workload of teachers as proxied by pupil-teacher ratios is 40:1 at the primary level and 23:1 at the secondary level which are comparable to other countries in the Sub-Saharan Africa region and other countries of the same income group; see Appendix Figure A2 (World Bank, 2023). Student completion rate is 64 percent at the primary level which is comparable to the regional average and average for the same income group, and 38 percent at the secondary level which is slightly lower than those of peer countries as reported in Appendix Figure A3 (World Bank, 2023). At both primary and secondary levels, completion rates of girls exceed those of boys.

### 2.3 Student learning in Burkina Faso

Students in the country still have low levels of learning. In an analysis of test scores of primary school students in 14 Francophone countries, only one in three early primary student in Burkina Faso has met what is considered minimum proficiency level in the language of instruction — the level at which students have gained the skills that would allow them to continue their schooling without difficulty (PASEC, 2020, Le Nestour, 2021). Only one in five students can count more than 80 (a skill usually gained by the second grade of schooling). In both language and math, students in Burkina Faso performed slightly lower than the mean across the countries in the sample. Given that teacher quality is directly associated with better learning outcomes—where approximately one-third of the variability in test scores in the surveyed countries can be attributed to variations in teacher knowledge (Bietenbeck et al., 2023) —the availability of teachers, especially knowledgeable and experienced teachers, is imperative to addressing these low learning levels.

## 3 Methodology

### 3.1 Data

We use the annual school census data in Burkina Faso’s EMIS for the analysis in this paper. The school census collects quantitative data on a range of school level indicators. We have data for six years, from 2014 to 2019. The data covers public, private and religious primary schools across the country. The total number of schools included in the census ranges from 13,200 in 2014–16,482 in 2019. A notable feature of the Burkina Faso school census is that it has a teacher roster which is used to collect demographic and employment data on all teaching staff in every school. The total number of teachers across all schools on whom data were collected ranges from 42,477 in 2014–65,651 in 2019.

A distinct feature that makes the data suitable to build individual attrition profiles for teachers is that there is a unique teacher identification (ID) that is, at least in theory, consistently used to identify teachers across various census years. Therefore, as long as the IDs are accurately captured, it should be possible to track teachers over time. The census captures data on all teachers employed at the school at the time of data collection, but it does not include any explicit data on attrition such as the total number of teachers who have left the school during the census year. Therefore, we constructed a new variable to indicate the attrition status of each teacher in the roster between two consecutive years. In other words, each teacher captured in the census data in year t is assigned one of three possible values for year t+1:

• “retained” if they remained in the same school in yeart+1,
• “switched school” if they are captured under a different school in yeart+1,
• “left the profession” if they are not captured under any school which is part of the census in yeart+1 or any subsequent year until 2019.

We used the combination of teacher ID, surname and date of birth to track teachers over census years. Once we have constructed attrition variables for each teacher, we have then computed–for each school–, the aggregate retention, the number of teachers who switched schools, and the number of teachers who left the profession. Since we have measured these indicators for all schools over multiple years, we are able to construct a panel of school-level attrition data covering 5 years.

### 3.2 Data limitations

Although the school census data is supposed to maintain a unique ID for each teacher on the roster, there is extensive data entry error which is common in administrative data that is not especially cleaned with a specific research objective in mind. Since the census data is not commonly used to construct teacher-level panel datasets, it appears that limited attention was given to the accuracy of the teacher ID data. Unfortunately, the name and date of birth of teachers captured in the roster are also subject to the same data entry inaccuracies which makes them inadequate for tracking teachers on their own. Therefore, we had to use teacher ID, surname and date of birth simultaneously to identify teachers and match their data across census years.

Both the individual-level and school-level attrition data are subject to some degree of measurement error due to the way the indicators were constructed using the raw data in the teachers roster. Firstly, teachers who, for any reason, were not captured in subsequent survey rounds while still being in the workforce are deemed to have atritted. Secondly, too many observations may have failed to match across census years due to the stringent requirement of identifying details that is used to track teachers and the likelihood of data entry error in either one of the three identifying variables. Both of the above factors may have led to an overestimation of aggregate attrition rates such that the rates we find could be higher than other national estimates of teacher attrition.

However, there is no reason to believe that this measurement error is correlated with any individual or school-level characteristics as it is mostly driven by data entry errors. In Table A2 in the Appendix, we compare the characteristics of teachers by the number of years they were tracked in the census data (regardless of attrition status). There is little systematic difference in the basic characteristics of teachers who were successfully tracked for the entire length of the panel compared to those tracked only for parts of it. As such, we do not anticipate that the measurement errors due to uneven tracking will affect our analysis of the attrition profiles of various groups of teachers or the correlates of teacher attrition at the school level.^2^

Another issue with the data is that the total number of schools in the census has increased more dramatically over the six-year period than could be explained by a plausible trend in the construction of new schools. The most likely explanation is that data collection coverage expanded significantly over time. But there is no systematic difference in type of schools (rural versus urban) or teacher characteristics between the pool of schools across the years, as the descriptive statistics in Table 1 show. Hence, the rapid increase in the number of schools in our data is unlikely to affect the analysis in Section 4.

**Table 1:** Descriptive statistics for the sample.

		**Year**					
	**Average**	**2014**	**2015**	**2016**	**2017**	**2018**	**2019**
Number of teachers	53,906	42,477	45,833	51,583	54,968	62,921	65,651
Average age (standard deviation in parentheses)	35.35	35.1	35.4	35.2	35	35.4	36
		(6.26)	(6.21)	(6.32)	(6.4)	(6.5)	(6.5)
Education							
% with junior high school	82 %	84 %	84 %	83 %	84 %	80 %	78 %
% with senior high school	14 %	12 %	13 %	13 %	14 %	15 %	17 %
% with tertiary education	3 %	3 %	3 %	3 %	3 %	3 %	4 %
Gender							
% female	46 %	42 %	44 %	47 %	48 %	49 %	49 %
Type of contract							
% civil service	57 %	19 %	16 %	14 %	97 %	99 %	99 %
% temporary contract	41 %	80 %	83 %	85 %	1 %	0 %	0 %
Number of schools	12,344	13,204	13,950	14,655	15,330	15,756	16,482
% rural	81 %	82 %	81 %	81 %	81 %	81 %	81 %
Number of violent conflict incidents by region*	5.9 (12.1)						
Regional Human Development Index**	0.428 (0.085)						

*Source:* Authors’ analysis of the underlying data as described in Section 3.

*Note*: The first column shows the average of the descriptive statistics from the six years in the dataset. The other columns represent the raw data from the surveys.

* Conflict data for the census years was compiled from Armed Conflict Location and Event Data (ALCED) dataset.

** Subnational HDI data for regions was obtained from the Global Data Lab.

### 3.3 Description of the sample

The dataset we use for this analysis contains 54,000 teachers per year in 12,300 primary schools spread across the 13 administrative regions in Burkina Faso (see Table 1 for the descriptive statistics of the sample). The average teacher age is 35 years old which is in line with other analyses of teacher demographics for the country (Evans and Mendez Acosta, 2023a; World Bank, 2022). Almost all of the teachers have completed at least some level of secondary education: over 80 percent of the teacher population has completed lower secondary education, while another 14 percent has completed upper-secondary education and a smaller number (3 percent) has attained college degrees. In general, teachers in Burkina Faso are considered competent—at least 88 percent of primary school teachers in the country have the minimum required qualifications to teach in primary education in 2022 (UNESCO, 2023). There are slightly fewer female teachers than male teachers (45 percent of the most recent count of teachers are female), and this is consistent for most regions except for the regions Cascades and Sud-oest where male teachers outnumber female teachers 3:2 (Appendix Table A1).

At the beginning of the period of our analysis (2014), only a fifth of the teacher workforce were permanent members of the civil service while the rest were on temporary contracts. At that time, contract teachers either had to pass a competency test or undergo a pre-service teacher training program to be eligible for a civil service position. However, beginning in January 2016, all contract teachers were granted civil service status (UNESCO, 2020), which is reflected in the census data that shows almost zero contract teachers in the database from 2016 onwards. The change in status may have had anticipatory effects on teachers’ attrition rate both for contract teachers (who might be more likely to stay given the imminent promotion) and for civil service teachers (who might be inclined to leave if the new teachers are viewed as competition). For these reasons, we forego the analysis on teacher retention rate by contract type.

Between 2014 and 2019, the number of schools in the survey have grown by 3200 schools (an increase of 25 percent over six years). Although there were several ad hoc programs of school construction (for example, a non-profit built 117 schools between 2014 and 2021 (buildOn, 2021)^3^), we find no evidence of a national school construction program that would explain this substantial increase in schools. The dataset itself also reports only around 900 schools founded between 2015 and 2019. In addition, while gross primary completion rates have slowly increased from 88 percent in 2014, up to 92 percent in 2018, and back to 90 percent in 2019, we would expect a higher jump in enrollment if there were that many new schools constructed over that time period (World Bank, 2023). More likely, this increase reflects improved response rate to the survey as more schools joined the annual census data collection.

**Fig. 1:**
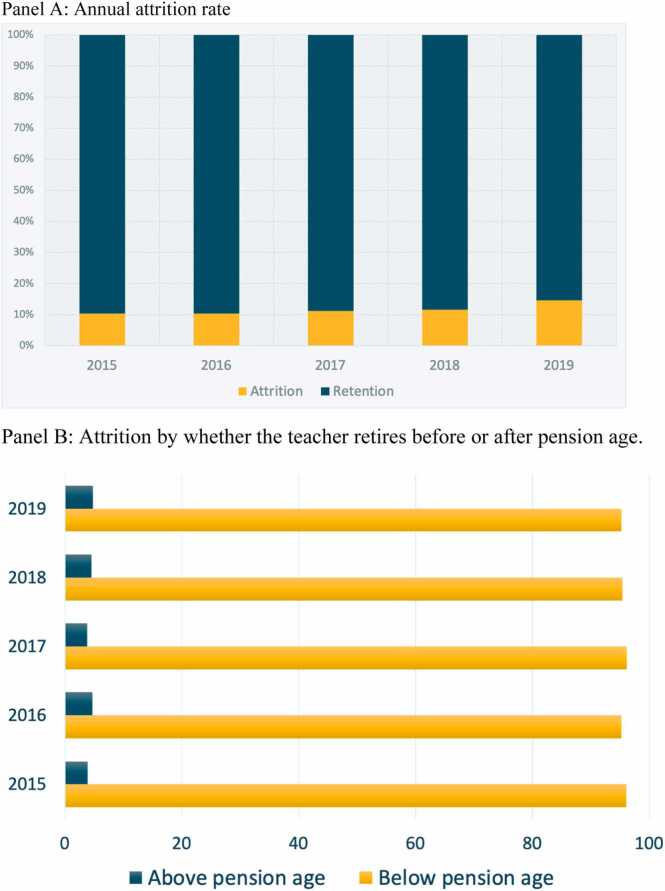
Teacher attrition rates among primary school teachers in Burkina Faso (2015–2019), Panel A: Annual attrition rate, Panel B: Attrition by whether the teacher retires before or after pension age.

Our final dataset shows, between 2015 and 2019, about 12 percent of teachers left the workforce (Fig. 1, Panel A). The first two years saw slightly lower rates of attrition of around 11 percent, potentially coinciding with the en masse promotion of teachers from contractual to civil service posts. Almost all of these teachers are younger than the prescribed retirement age of 60 years old when primary school teachers start receiving their pension (Fig. 1, Panel B). The attrition rate we calculate is similar to the 11 percent that Zeitlin (2021) finds in Rwanda, the only other study we know of that uses EMIS data to estimate teachers’ attrition in a Sub-Saharan Africa context. However, we note that missing data and the difficulty of tracking teachers from year to year in our dataset as outlined in Section 3.2 limit the representativeness of these attrition rates such that actual teacher attrition at the national level is likely to be lower than what we see from the dataset.

### 3.4 Empirical method

We examine the correlates of attrition both at the individual and school levels. At the individual level, we seek to analyze how the propensity to attrite changes over the lifecycle of individuals for groups of teachers with different demographic and socioeconomic characteristics. To do this, we adopt the Age-Period-Cohort framework, a widely recognized method for disentangling the intertwined effects of age, time period, and birth cohort on a particular phenomenon. The method enables us to describe the age profile of teachers’ attrition while also taking into account the potentially heterogeneous attrition profiles of different cohorts at the same age. Moreover, it would also help us to disentangle the time effects that are common to all age groups and birth cohorts and are possibly caused by exogenous factors. More importantly, we would be able to describe the age, cohort and period patterns in the attrition of various salient groups such as male and female, teachers with different levels of education, and teaching staff with various roles.

The longstanding challenge in implementing this model is the difficulty of identifying the age, cohort and period elements statistically because the value of cohort is completely determined by age and period (i.e. cohort=period−age). We employ the Age-Period-Cohort-Interaction (APC-I) approach proposed by Luo and Hodges (2020) to estimate the age and period effects of teachers’ attrition with the cohort effects represented as age-period interaction. This means the cohort effect can be identified because it is estimated indirectly without imposing additional restrictions to circumvent the collinearity issue. Specifically, we estimate the following model:

(1) $$g(E(Y_{\mathrm{ij}}))=\mu +\alpha_{i}+\beta_{j}+{\alpha \beta }_{\mathrm{ij}(k)}$$

Where $E(Y_{\mathrm{ij}})$ represents the expected value of teachers’ attrition for the *i*th age group at time *j* while *g* is the link function. α denotes the age effect whereas β stands for the period effect. ${\alpha \beta }_{\mathrm{ij}(k)}$ denotes the interaction of the *i*th age-group and *j*th period, corresponding to the effect of the *k*th cohort.

Since individual teacher attrition is a binary variable, the APC-I model in (1) can be estimated using a binary logit model given bellow:

$$\ln\left(\frac{\pi }{1-\pi }\right)=\mu +\alpha_{i}+\beta_{j}+{\alpha \beta }_{\mathrm{ij}(k)}$$

The second level of analysis consists of estimating the correlates of school-level attrition. We employ standard panel data techniques to estimate the predictors of teacher attrition either in the form of switching school or entirely leaving the profession. The underlying specification takes the form of a standard panel data model with time-invariant variables:

(2) $$y_{\mathrm{it}}={X\prime }_{\mathrm{it}}\beta +Z_{i}\gamma +\mu_{i}+\varepsilon_{\mathrm{it}}$$

Where $y_{\mathrm{it}}$ is teacher attrition rate at school *i* in year *t*, $X_{\mathrm{it}}$ is a vector of time varying covariates and $Z_{i}$ is cross-sectional time-invariant variables. Estimating the coefficients in (2) using a standard fixed effects estimator would remove any time-invariant characteristics of schools which we expect to predict teacher attrition, such as geographic location and public or private ownership. Therefore, we employ the Hausamn-Taylor estimator which would enable us to recover the coefficients of some of the time-invariant variables at the same time as accounting for unobserved individual heterogeneity (Hausman and Taylor, 1981).

## 4 Results

### 4.1 Attrition rates by personal characteristics

**Fig. 2:**
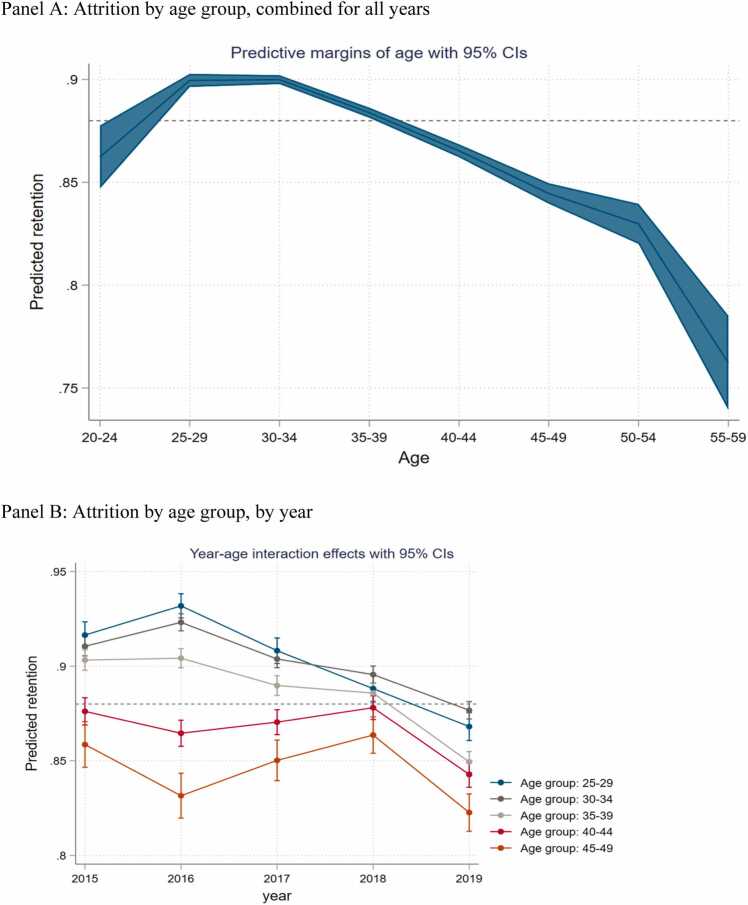
Teacher attrition rates among primary school teachers in Burkina Faso by age group, Panel A: Attrition by age group, combined for all years, Panel B: Attrition by age group, by year, Note: The dashed line shows the average retention for the whole group. Age group 20–24 is the reference category.

Age. Younger teachers in the age group of 25–35 years are the most likely to remain in the workforce with a retention rate of 90 percent (44 percent higher odds of staying than the reference age group 20–24, p-value < 0.01, Table 2 column 1). Teachers above 39 years old see progressively lower rates of retention (Fig. 2, Panel A). There is a decline in retention from 83 percent for teachers age 50–54 to 77 percent for teachers age 55–59, signaling early retirement for teachers in this cohort. The trend—where retention starts relatively low for the first five years in teaching, peaks for teachers with 5–15 years of experience and then steadily declines henceforth—is true for any year between 2015 and 2019 (Fig. 2, Panel B). The lower than average retention rates in 2019 could potentially be attributed to a combination of factors. Firstly, the 2019 data does not benefit from the presence of data in subsequent years which could be used to verify whether missing data is due to actual attrition or one-off data entry error in that round. Hence, attrition rates for 2019 are likely to be overstated. Secondly, the escalation of the conflict that had broken out first in 2016 and continued to escalate in later years may have contributed to higher attrition of teachers (Seibert, 2020). The higher exit rate for early career teachers is consistent with what we see in Rwanda where 40 percent of teachers in their first years resign their posts and attrition remains above 10 percent in the first five years of teaching (Zeitlin, 2021), and opposite to what we see in Kenya where older teachers (albeit, secondary school teachers) are more likely to attrite (Mabeya 2021).

**Table 2:** Demographic and professional determinants of teacher retention.

	(1)	(2)	(3)	(5)	(6)
*Base reference (Age: 20–24)*					
Age: 25–29	1.44***				1.44***
	(0.10)				(0.10)
Age: 30–34	1.45***				1.44***
	(0.10)				(0.10)
Age: 35–39	1.23***				1.19***
	(0.08)				(0.08)
Age: 40–44	1.03				0.98
	(0.07)				(0.07)
Age: 45–49	0.88*				0.83***
	(0.06)				(0.06)
Age: 50–54	0.79***				0.74***
	(0.06)				(0.06)
Age: 55–59	0.53***				0.50***
	(0.05)				(0.05)
Female		0.89***			0.92***
		(0.01)			(0.01)
*Base reference (Educational attainment: Junior high school)*					
Senior/technical high school			1.09***		1.05***
			(0.02)		(0.02)
Tertiary education			0.80***		0.83***
			(0.03)		(0.03)
*Base reference (Personnel type: Assistant teachers)*					
Regular teachers				1.39***	1.40***
				(0.03)	(0.03)
School Principals				1.18***	1.11***
				(0.02)	(0.02)
Constant	7.16***	9.13***	8.84***	7.14***	6.66***
	(0.48)	(0.16)	(0.14)	(0.16)	(0.47)
Year fixed effects	Yes	Yes	Yes	Yes	Yes
Observations	266,299	266,299	262,297	265,944	261,947

Note: The table shows the odd ratios of a teacher staying in the workforce given several demographic and professional factors. All specifications include year fixed effects. Robust standard errors (in exponentiated form) in parentheses. *** p < 0.01, ** p < 0.05, * p < 0.1

**Fig. 3:**
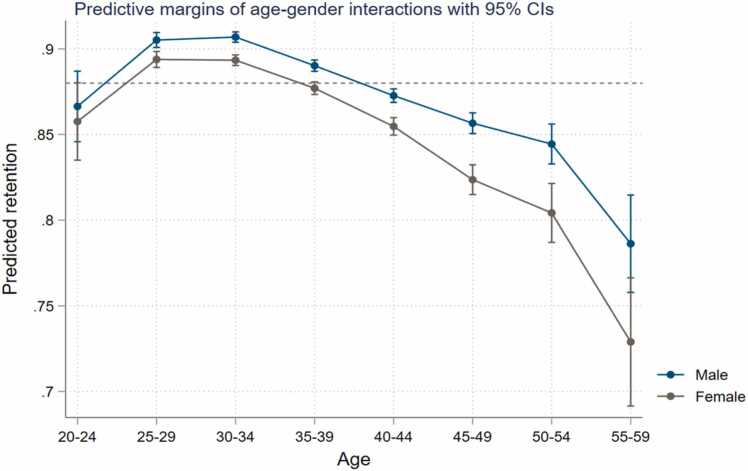
Teacher attrition rates among primary school teachers in Burkina Faso by gender.

*Gender.* Female teachers have 11 percent lower odds of staying in the public teacher workforce than male teachers on average (p-value < 0.01) (Table 2, column 2). When gender is plotted against age based on the age-period-cohort estimates, we see that female teachers are more likely to leave at every age group, and the gender gap widens for teachers older than 40 years old (Fig. 3). Particularly, the widening gap over the course of the 40 s might suggest that women are more likely to drop out of the workforce than men as family responsibilities accumulate. In Sub-Saharan Africa, women cite unpaid care work as the reason for not participating in the workforce (34 percent) (Addati et al., 2018). For women aged 40 years and above, this unpaid care may take the form of caring for aging parents and young grandchildren rather than their own children^4^ and may mean moving to part time work or the informal sector. This finding is opposite to what we see in Rwanda (Zeitlin, 2021) and Kenya (Mabeya 2021), suggesting that the dynamics of gender affect job choices differently for Burkina Faso.

**Fig. 4:**
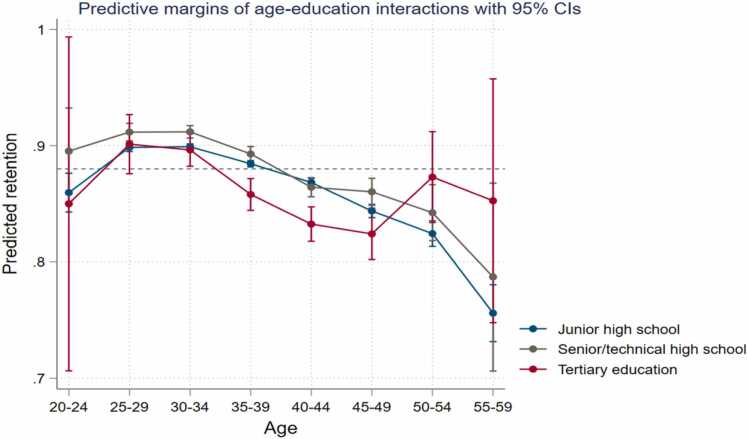
Teacher attrition rates among primary school teachers in Burkina Faso by educational attainment.

*Education.* Teachers with university level education have 20 percent lower odds of staying in the workforce on average (p-value < 0.01), especially in the earlier years, compared to teachers with only junior high school (Table 2, column 3; and Fig. 4). Interacted with age, we find this trend to hold true across the age groups except for teachers 50 years and older, when teachers with the highest education levels are most likely to be retained (Fig. 4).

**Fig. 5:**
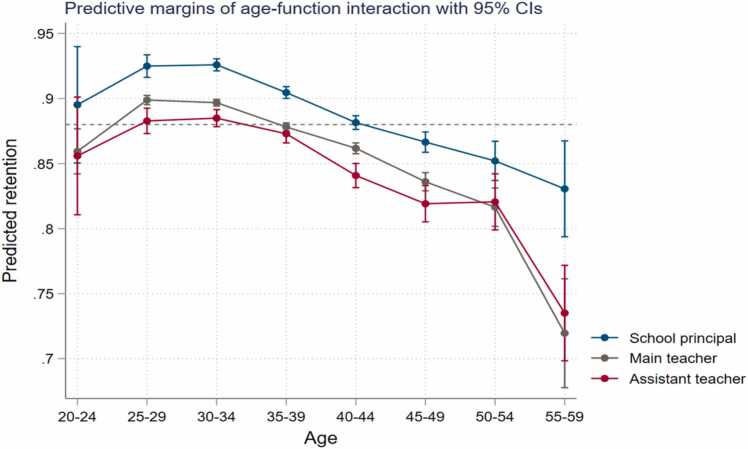
Teacher attrition rates among primary school teachers in Burkina Faso by function.

*Function.* Assistant teachers who are below regular teachers in the career ladder are more likely to leave than the regular teachers (odds ratio of regular teachers staying compared to assistant teachers = 1.39, p-value < 0.01) except for the oldest age group (55 years and up). This is likely because main teachers leave the workforce to pensionable early retirement while assistant teachers may find financial incentives to stay active in the workforce (Fig. 5). School principals enjoy the highest rate of retention across all ages which suggests that the usually higher salary and benefits, elevated social standing, and the non-teaching tasks associated with management positions make staying attractive.

Taken together, we see that teachers in the first five years of their career and teachers with more advanced educational attainment—precisely the teachers that may have access to better paying opportunities either outside the education sector or outside the civil service—have a relatively high likelihood of leaving. Conditional on not leaving early in their careers, teachers with tertiary degrees often have high retention in their later years, which coincides with the higher retention of teachers in higher management positions (positions that often require more advanced degrees). This suggests that highly qualified teachers are more likely to stay given access to promotions. Female teachers in Burkina Faso are more likely to leave than male teachers contrary to the evidence from Rwanda (Zeitlin, 2021) and in the majority of LMICs (Le Nestour and Moscoviz, 2020).

One classic explanation to higher female teacher attrition is related to family rearing when women leave the workforce to (temporarily) raise children.^5^ In our sample, male teachers are twice more likely than female teachers to complete upper-secondary school and have tertiary degrees (both genders are equally likely to complete lower-secondary education) and only 10 percent of school principals are women. This points to limited career progression for female teachers in the teacher workforce, such that they may be the parent of choice to stay at home. However, regressing probability of retention with age, gender, educational attainment, and position shows that the gender influence is still statistically significant beyond these factors. We are unable to verify either the parental status of leaving teachers or to track teachers who leave and return to the service, either of which can indicate evidence for female teachers exiting the workforce to raise children.

### 4.2 Attrition rates by school characteristics

**Fig. 6:**
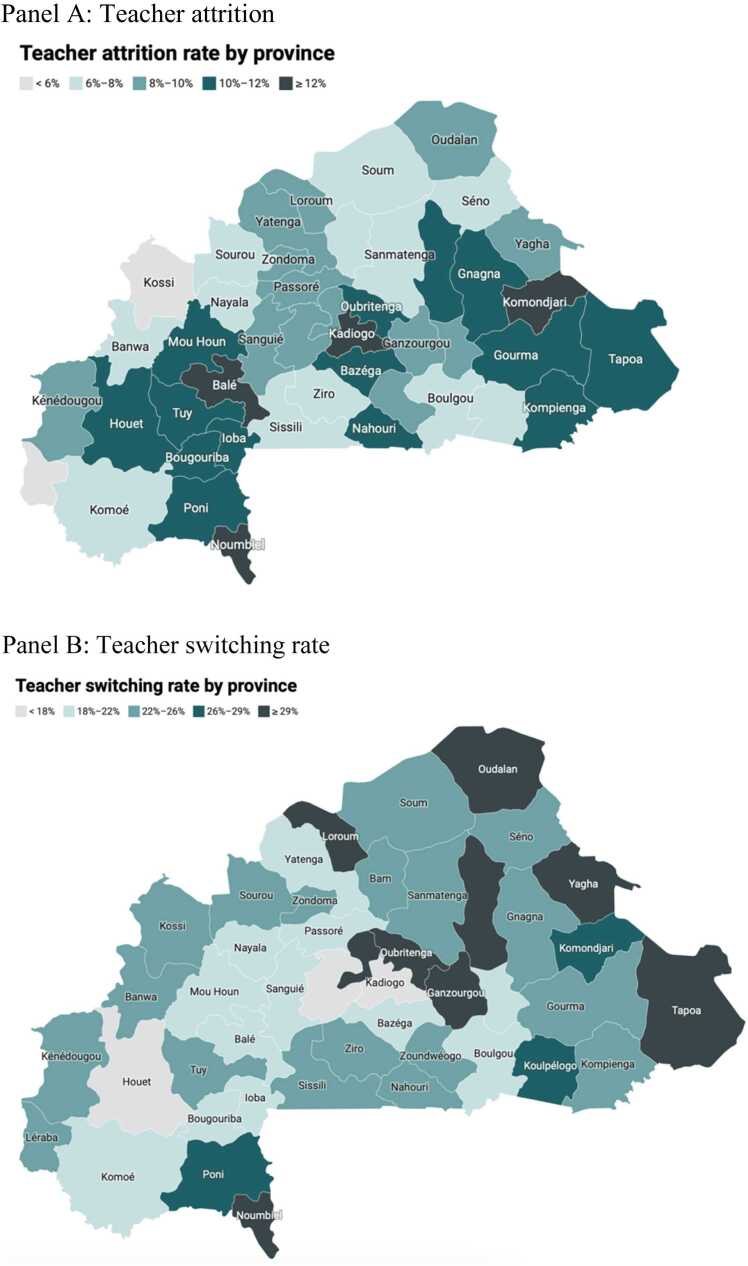
Teacher attrition and switching rates by province, Panel A: Teacher attrition, Panel B: Teacher switching rate, Note: The rates displayed in the figures are simple averages of school-level attrition and switching rates for each administrative province.

We also report school-level correlates of the annual attrition rate (share of teachers leaving the profession) and annual school-level turnover (share of teachers switching schools) (Table 3). We show province level teacher attrition and switching rates in Fig. 6. For the education system as a whole, the relevant metric is the proportion of teachers who leave the workforce entirely. However, for a particular school, what really matters as a measure of attrition is the proportion of teachers who leave the school whether they are leaving the profession or transferring to another school. Therefore, we estimate the predictors of both forms of attrition to obtain a complete picture of which types of schools are likely to be affected by teacher turnover.

**Table 3:** Determinants of school-level teacher attrition.

	Percentage left profession	Percentage left profession	Percentage switched school	Percentage switched school	Percentage switched school (Male)	Percentage switched school (Male)	Percentage switched school (Female)	Percentage switched school (Female)
Urban school	1.41***	−0.348	−12.1***	−10.0***	−10.11***	−7.78***	−1.35	−2.11 * *
	(0.406)	(0.869)	(0.716)	(0.733)	(0.914)	(0.912)	(0.93)	(0.921)
Regional Human Development Index		31.0**		−147.3***		−125.0***		−50.0 * **
		(14.9)		(9.42)		(10.3)		(10.3)
Incidence of violent conflict in province (log)	1.57***	1.51***	−3.17***	−2.48***	−4.07***	−3.47***	−3.19***	−2.81
	(0.179)	(0.183)	(0.297)	(0.314)	(0.326)	(0.34)	(0.324)	(0.335)
Public school dummy	−10.7***	−9.11***	3.03***	−1.64	1.78	3.7	31.1***	32.2 * **
	(0.728)	(0.936)	(1.44)	(1.46)	(2.41)	(2.36)	(2.97)	(2.87)
Controls	School infrastructure, school age, school management committee, share of teachers over 40 years old							
Estimation method	Hausman-Taylor							
Region dummies	Yes	No	Yes	No	Yes	No	Yes	No
Observations	42,072	42,072	39,355	39,355	35,987	35,987	26,967	26,967

Note: Robust standard errors in parentheses. *** p < 0.01, ** p < 0.05, * p < 0.1

We find that teachers are more likely to leave the profession from schools with higher provincial incidence of violent conflict.^6^ A one standard deviation change in conflict incidence is associated with an 18 percent increase in the share of teachers who leave the workforce from a given school. Similarly, teachers employed in urban schools are more likely to leave the profession than teachers in rural schools. Specifically, attrition in urban schools is 1.14 percentage points (0.09 standard deviation) higher than attrition in rural schools. The coefficient of urban school becomes insignificant, however, when we include an indicator for the level of regional development. Teachers based in more developed provinces, as measured by the regional human development index, are more likely to leave the profession than teachers in less advanced areas. This suggests that urbanization may be standing in as a proxy for level of development which drives attrition by increasing the availability of more lucrative labor market options other than teaching. Taken together, the results with respect to the conflict and regional development dimensions of school location indicate that teacher attrition is partly governed by an interplay of push and pull factors. The map in Panel A of Fig. 6 shows that the province hosting the capital, Ouagadougou, is one of the regions characterized by the highest percentage of teachers leaving the workforce.

The other factor that might predict the percentage of teachers who are leaving the workforce is the type of school ownership. Around 1 in 4 of the primary schools in our panel dataset is either a private or religious school whereas the other 3 are public schools. The results in Table 3 show that teachers in public schools are almost 10 percent less likely to leave the profession than teachers in private or religious schools. This result is not surprising because teachers are more likely to stay in government funded schools which are historically better endowed than private institutions. In addition to endowment, public schools might be better managed which, in turn, attract higher skilled and more motivated teachers (Leaver et al., 2019). Except for a few elite private schools in large cities, private low-fee schools often have less favorable employment conditions for teachers.^7^

What type of schools do teachers transfer away from? Unsurprisingly, rural schools and schools in less developed regions all see statistically significant higher rates of teacher switching. This means that teachers tend to transfer away from rural and remote schools to urban schools in more developed regions before they leave the teaching workforce, presumably for greener pastures such locations may offer. This trend of career progression is confirmed by a detailed study of the employment histories of a sample of teachers in Burkina Faso which shows that entry-level teachers often start in rural areas before gradually moving to small towns and larger cities as part of the internal transfer process (Sirois, 2017). The map in Panel B of Fig. 6 shows that provinces in the northern part of the country tend to experience a higher rate of teachers switching schools.

It is curious that schools situated in high-conflict regions exhibit lower rates of teacher-switching compared to those in relatively safer areas. One way of interpreting this finding is that teachers in conflict-affected areas are more likely to leave the workforce and the respective area altogether, as confirmed by the previous results, than trying to switch schools. It could also be the case that administrative structures that would normally be responsible for teacher transfer processes are affected by the conflict that switching schools through the internal transfer process is less frequent in conflict-affected areas. Public schools are also more likely to see lower levels of teacher switching schools, but the effect is not precise.

Male teachers are more sensitive to these school correlates than female teachers: for example, male teachers are up to three times more likely to transfer away from schools in rural areas (-7.78 percentage points decrease in probability to switch schools for male teachers given that the school is in an urban location versus −2.11 percentage points for female teachers) and twice more likely to do so for schools with lower regional development index (10 percentage points versus 4 percentage points per standard deviation). The incidence of violence in the province is a strong predictor for switching schools for male teachers but not for female teachers. Opportunity rather than preference may explain the gender gap in the decision to switch: male teachers are more likely to have higher educational attainment and hold higher teaching positions, allowing them to more easily switch schools than their female peers.

## 5 Policies for improving teacher retention

The descriptive nature of this study allows us to profile teachers most likely to attrite according to several demographic factors (age, gender, tenure, and qualifications). In the absence of additional information on their motivations, such as qualitative surveys, discrete choice experiments, or impact evaluations of interventions, we are not able to report on the exact mechanism of decision-making for these teachers. We therefore rely on the established literature that propose conceptual frameworks to understand teacher attrition (UNESCO, 2010, Nguyen, 2020, See et al., 2020) to draw potential links between the teacher attrition profiles and what might be driving these teachers to quit (Table 4). The high attrition among young teachers early in their careers, teachers with tertiary education, and those in currently low positions suggest that factors such as compelling opportunities outside of teaching combined with potential unsatisfactory working conditions and professional growth may affect the decision to attrite. These drivers then can be linked to corresponding policies that improve teacher working conditions and benefits to be at par with other competing opportunities. Similarly, the higher likelihood of attrition for female teachers suggests a gender-driven difference in working conditions and/or social and cultural expectations, which require policies that specifically overcome these barriers to retention.

**Table 4:** Profiles of teachers most likely to attrite in Burkina Faso, potential factors that drive attrition, and suggested policy solutions.

Profile of teachers who are likely to attrite in Burkina Faso	Type of teacher attrition driver drawing from earlier works on the conceptual framing of teacher attrition (UNESCO, 2010, Nguyen, 2020, See et al. 2020)	Potential policy solutions
Early career teachers (first five years of teaching)	- Opportunities outside of teaching - Dissatisfaction with working conditions/professional growth	*Policy recommendation #1:* Financial and non-financial incentivesPolicy recommendation #2: Opportunities for teacher professional development and improved teacher working conditionsPolicy recommendation #3:Building on motivations and improving overall job satisfaction
Teachers with tertiary education		
Teachers with lower positions		
Female teachers	- Gender gap in working conditions - Personal factors, social dynamics and expectations	*Policy recommendation #4:* Efforts to recruit female teachers

In this section, we discuss potential policy options in addressing teacher attrition and the scope of evidence behind these interventions. Across these policy options, only the monetary incentives have a robust evidence base as shown by a few systematic reviews in the topic (Evans and Mendez Acosta, 2023b; See et al., 2020). The other policies, while promising in the contexts in which they were implemented, have more limited evidentiary support in diverse contexts. Nevertheless, we believe there is still merit in expanding the tools policymakers may use to improve retention to include these measures (as may be appropriate in their contexts), given the interaction of personal, financial, and structural factors affecting teacher attrition as discussed in Section 2.

### 5.1 Financial and non-financial incentives

Providing financial benefits and related perks such as housing, transportation, and allowances is the most straightforward to implement and also the most evaluated policy option in improving teacher retention especially for particularly hard-to-staff schools or schools that see higher turnover (Evans and Mendez Acosta, 2023b; See et al., 2020). Most of the evidence for financial incentives point to positive outcomes on attracting and keeping teachers, but results vary depending on implementation, and the effectiveness of these programs may only last as long as the incentives are present. An incentive of up to 26 percent increase in salary for teachers working in poor neighborhoods in Uruguay improved retention for teachers but only for those later in their careers such that the average teacher tenure increased by one year for beneficiary schools (Cabrera and Webbink, 2020). A similar program that provided a wage premium of between a quarter to a third of current salaries improved teacher turnover in Brazil by 5 percentage points (Camelo and Ponczek, 2021). Another incentive program, although of lesser monetary value and this time in Chile, did not change teacher retention but in fact reduced hours worked by teachers (Hinze-Pifer and Méndez, 2016). The evidence from the Sub-Saharan Africa region shows mixed results: a 20 percent rural hardship allowance in Zambia led to more teachers but no effect on average student scores (Chelwa et al., 2019), and a similar hardship allowance of 30–40 percent in Gambia led to 10 percentage point more (and better qualified) teachers in remote areas (Pugatch and Schroeder 2014), but with also no effect on average test scores (Pugatch and Schroeder 2018).

As countries often have different teacher pay contexts (how teacher pay compares to other professions in the country and how that pay adequately provides for an acceptable standard of living within the local context), financial incentives may have different mileage in changing exit behavior. Since Burkina Faso’s teachers are relatively well paid compared to other similarly educated professionals, at least for teachers who have secondary education, financial incentives may work best when targeting teachers either in the first five years of teaching (when probability of attrition is high) or those with higher educational attainment, potentially tied with opportunities for professional development or access to management positions.

The current wage structure for teachers in Burkina Faso allows for government-provided housing for teachers assigned to schools in rural areas, and a housing allowance to compensate if there are no appropriate accommodations available, in addition to a “hardship allowance”, a special allowance based on the poverty zone of the school. All of these bonuses can represent up to 25 percent of a new teacher’s salary (Sirois, 2017). However, teacher personal preferences may also differ (e.g. teachers from those rural regions may require less incentive to teach there because of family ties) such that incentives might be more cost-effective if they are also designed to respond to teacher preferences and not just on rigid school-level characteristics (Bobba et al., 2022).

### 5.2 Opportunities for teacher professional development and improved teacher working conditions

There is far less evidence on improving retention through teacher professional development, and those that are available vary in outcomes, but the particularly successful programs, at least in terms of improving student outcomes, often link participation in professional development programs to incentives such as promotions (Popova et al., 2022; Bennell and Akyeampong, 2007, Quota and Bhatia, 2022). A survey of over 130 public expenditure reports across low and middle income countries find one fifth of the reports mention in-service trainings, often in the context of their limited availability and the gap in participation in these trainings by female teachers and those from poorer communities (Evans and Mendez Acosta, 2023c). As such, improving in-service trainings, through coaching and mentoring, structured pedagogy, and appropriate use of technology aids, may help improve the quality of existing teachers and make career promotions more accessible and equitable.

Given that teachers with management positions (such as school principals) are much more likely to be retained than regular teachers in Burkina Faso, ensuring access to teacher professional development, especially those tied to promotion, may help encourage retention for qualified teachers especially those with tertiary education who are more likely to attrite. Similarly, ensuring ample opportunities for promotion and professional development for early career teachers may help mitigate the high attrition rate for teachers in their first five years in the profession.

### 5.3 Building on motivations and improving overall job satisfaction

**Figure A4:**
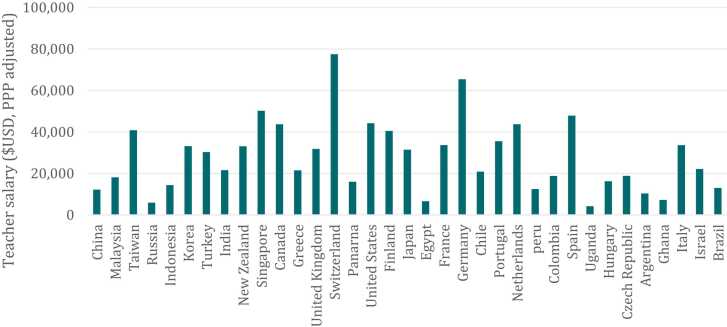
Index ranking of countries according to social prestige of teachers against teacher salary in that country., Source: Authors’ figure from data from Global Teacher Status Index 2018 (Dolton et al., 2018), Note: Countries are listed from highest social prestige (left, China) to lowest social prestige (right, Brazil). The trendline displayed is a linear estimation of the relationship between the two variables

A synthesis of over 200 studies globally on who becomes a teacher and why documents the presence of intrinsic and altruistic motives to teaching in addition to extrinsic motivations (See et al., 2022). For the group that would have gone to teaching but decided not to, the social status of the profession is rated as important, alongside the working environment and salary (See et al., 2022). High-salary and social prestige do not necessarily go together: the Global Teacher Status Index ranks countries based on the society’s respect to teachers, and there is no clear indication that countries that pay high teacher wages respect them more (see Appendix Figure A4).

Changing social perceptions is less straightforward than increasing salaries, but there is some evidence that low-cost behavioral nudges may have an impact on tapping into teachers’ altruistic motives. For example, a nationwide teacher recruitment campaign in Peru encouraged teachers to apply for more disadvantaged/remote schools (Ajzenman et al., 2024). They sent out messages and pop-outs in the online application portal either highlighting the altruistic nature of teaching (i.e. “Thank you for being an agent of social change”) or promoting an existing monetary incentive when they apply to disadvantaged schools. Both interventions improved applications to remote schools, especially for high-performing teachers that received the “altruistic” messages. A similar program in Chile had the opposite effect where high-performing students are less likely to be attracted to teaching when primed with “altruistic messages,” while low-performers (ostensible from disadvantaged households) are more likely to respond to messages promoting monetary rewards to teaching (Ajzenman et al., 2021).

Neither motivation to teach nor job satisfaction is included in our analysis as determinants of attrition (since our study is based on administrative data), but these factors are likely candidates in influencing the decision to switch or leave the profession for future research.

### 5.4 Efforts to recruit female teachers

Female teachers encourage enrollment and improve student learning outcomes especially for girls by serving as role models and encouraging higher aspirations (Muralidharan and Sheth, 2016, Haugen et al., 2014, Eble and Hu, 2020, Lim and Meer, 2020). However, female teachers are historically difficult to attract and retain in remote schools where they may be needed the most because of the lack of infrastructure such as housing and transportation or concerns over their personal safety in areas of conflict (UNESCO, 2019). In addition to policies that address the general teacher working conditions that affect all teachers, efforts that specifically address the barriers female teachers face may help improve teacher gender parity. Surveys of female teachers in Ghana and Kenya report that incentives such as study leave with pay, expedited promotion, provision of housing, and hardship allowance may make remote schools more attractive for teaching (Gad, 2015, Kamere et al., 2019). Recruiting teachers locally also has the advantage of potentially keeping female teachers in the workforce even as they raise families since they are more likely to have strong social support in their families within the local community. These policy interventions would particularly be helpful in the case of Burkina Faso where female teachers have much lower retention rate than male teachers.

## 6 Conclusion

Teacher attrition can be a significant challenge particularly if it leads to the exit of effective teachers and in light of the increasing teacher demand driven by growing school population in most low- and middle-income countries such as Burkina Faso. A nuanced understanding of the characteristics of the teachers who are likely to leave and the schools that are more prone to suffer from attrition is an important step in addressing teacher attrition. Surprisingly, there has been very limited effort so far to utilize administrative data, such as annual school census data, to study teacher attrition in regions such as Sub-Saharan Africa. This paper has used six years of school census data to examine the demographic and geographic correlates of teacher attrition in Burkina Faso.

In line with the literature on competing employment opportunities outside of teaching and family priorities, we find that younger teachers in their first five years of teaching, teachers with lower positions, and teachers who have completed tertiary education are more likely to quit the profession. We also find that female teachers are more likely to leave than male teachers, suggesting gender as a crucial factor in job choices. In terms of school characteristics, rural and remote schools are more likely to lose teachers to other schools while schools in more developed areas — ostensibly with more active industries and job markets — tend to lose teachers to options outside of teaching.

The findings suggest the need for teacher retention policies to especially target women and young teachers who are in the first few years of their careers. Additionally, the drivers of attrition and the destinations of teachers leaving schools in urban and more developed areas are likely to be different from those in rural and remote locations. Accordingly, the policy options to address attrition may also need to be differentiated based on the specific needs of various geographic areas and settings. As localized conflict and fragility continue to compound the effects of existing disadvantages on teacher attrition in certain areas, it is even more urgent to devise policy solutions that take these demographic and geographic factors into account.

## Funding

This work is made possible through funding from the 10.13039/100000865Bill and Melinda Gates Foundation.

## CRediT authorship contribution statement

**Amina Mendez Acosta:** Writing – review & editing, Writing – original draft, Visualization, Investigation, Data curation. **Bedasso Biniam:** Writing – original draft, Supervision, Methodology, Data curation, Conceptualization.

## Conflicts of interest statement

The authors declare no competing interest.

## Figures and Tables

**Appendix Table A1:** Descriptive statistics by region

	**Number of schools**	**Number of teachers**	**Proportion of female teachers**	**Proportion of schools considered to be in a rural location**	**Teacher attrition rate**	**Teacher attrition rate (female teachers)**	**Teacher attrition rate (male teachers)**
BOUCLE DU MOUHOU	1382	7076	41 %	92 %	8 %	6 %	6 %
CASCADES	658	3072	39 %	89 %	6 %	5 %	4 %
CENTRE	1850	5810	51 %	28 %	13 %	10 %	9 %
CENTRE-EST	1217	6443	51 %	82 %	8 %	6 %	6 %
CENTRE-NORD	1375	5842	45 %	91 %	8 %	6 %	5 %
CENTRE-OUEST	1283	8019	45 %	90 %	9 %	6 %	6 %
CENTRE-SUD	670	3875	49 %	83 %	10 %	7 %	7 %
EST	1267	6787	44 %	92 %	11 %	8 %	7 %
HAUTS-BASSINS	1538	7099	43 %	68 %	10 %	8 %	7 %
NORD	1654	7562	45 %	91 %	9 %	6 %	6 %
PLATEAU CENTRAL	967	4043	50 %	93 %	9 %	6 %	6 %
SAHEL	1037	4936	40 %	95 %	8 %	6 %	5 %
SUD-OUEST	858	4533	39 %	90 %	11 %	7 %	7 %
ALL REGIONS	15,756	75,097	45 %	81 %	9 %	7 %	6 %

*Note:* Attrition rates are regional averages of school-level attrition and represent attrition rates for five years (2014–2018).

**Appendix Table A2:** Descriptive statistics by tracking status

	**Number of appearances in matched dataset (number** **of years a teacher was tracked)**					
	**1**	**2**	**3**	**4**	**5**	**Average**
Number of teachers	29670	28731	14336	16044	25651	53,906
Average age	35.74	34.48	33.67	35.22	37.06	35.35
Education						
% with junior high school	80.59	82.45	82.2	83.35	82.85	82
% with senior high school	15.97	14.27	14.68	13.72	13.88	14
% with tertiary education	3.45	3.28	3.12	2.94	3.27	3
Gender						
% female	45.54	52.08	49.63	47.5	46.02	49
School location						
% rural	77.39	81.27	85.26	83.04	78.79	81

## Footnotes

1 The negative impacts of attrition on learning would most likely depend on the quality of teachers who leave. An incentive program in Peru encouraged teachers to move to hard-to-staff schools, improving retention by up to 5 percentage points. But while test scores in the schools with the incentive did not change, test scores in neighboring schools with no incentive (and which experienced higher attrition) saw up to 0.30 standard deviation higher math and test scores (Castro and Esposito, 2022). This suggests that the teachers who decided to move schools are lower-skilled such that the non-incentive schools were left with higher average teacher quality, if slightly fewer teachers. In many settings like Rwanda, teachers who leave because of better job opportunities are exactly those with desirable teacher qualities (higher educational attainment, specialized skills in STEM, or better mastery of language such as English) such that teacher loss conceivably results in a drop in test scores.

2 The measurement error seems higher for 2019 since the attrition rate for that year is higher than the average for the earlier four years by 3.6 percentage points. So, we tried to test the sensitivity of our findings in Section 4 to potential measurement error by excluding 2019 from the data. There is no significant difference in the results.

3 Another well documented program built over 120 girl-friendly schools over 10 years starting in 2006, but most of the school construction activities happened before the time period of our analysis (Ingwersen et al. 2019).

4 Median age of women in Burkina Faso on the birth of their first child is 20 years old (INSD and ICF. 2021). At 40 years old, women are more likely to bear the burden of caring for both elderly parents and their grandchildren especially given the importance of grandparents’ role in childcare in the region (Hatch and Posel, 2018, Mhaka-Mutepfa et al., 2017)

5 For example, a study in rural Pakistan reports that more than a quarter of female teachers plan to retire from teaching after getting married and almost half plan to stop if their spouses ask them to stop teaching to care for their families (de Talancé 2017).

6 The types of violence conflict include political violence, demonstrations and related strategic developments such as battles, explosions or remote violence, violence against civilians with or without reported fatality, riots, looting or property destruction, and related events as reported in the Armed Conflict Location & Event Data Project (ACLED, 2024).

7 In India, private school teachers earn less than a sixth of what civil service teachers do (Muralidharan and Sundararaman, 2013), and in Kenya, teachers of the same qualifications earn twice as much working for the public service than the private sector (Barton, Bold, and Sandefur, 2017).
